# Deep learning based on ultrasound images assists breast lesion diagnosis in China: a multicenter diagnostic study

**DOI:** 10.1186/s13244-022-01259-8

**Published:** 2022-07-28

**Authors:** Yang Gu, Wen Xu, Bin Lin, Xing An, Jiawei Tian, Haitao Ran, Weidong Ren, Cai Chang, Jianjun Yuan, Chunsong Kang, Youbin Deng, Hui Wang, Baoming Luo, Shenglan Guo, Qi Zhou, Ensheng Xue, Weiwei Zhan, Qing Zhou, Jie Li, Ping Zhou, Man Chen, Ying Gu, Wu Chen, Yuhong Zhang, Jianchu Li, Longfei Cong, Lei Zhu, Hongyan Wang, Yuxin Jiang

**Affiliations:** 1 Department of Ultrasound, Peking Union Medical College Hospital, Chinese Academy of Medical Sciences and Peking Union Medical College, No.1 Shuai Fu Yuan, Dong Cheng District, Beijing, 100730 China; 2 Department of Medical Imaging Advanced Research, Beijing Research Institute, Shenzhen Mindray Bio-Medical Electronics Co., Ltd., Beijing, China; 3 Department of Ultrasound, The Second Affiliated Hospital of Harbin Medical University, Harbin, China; 4 Department of Ultrasound, The Second Affiliated Hospital of Chongqing Medical University and Chongqing Key Laboratory of Ultrasound Molecular Imaging, Chongqing, China; 5 Department of Ultrasound, Shengjing Hospital of China Medical University, Shenyang, China; 6 Department of Medical Ultrasound, Fudan University Shanghai Cancer Center, Shanghai, China; 7 Department of Ultrasonography, Henan Provincial People’s Hospital, Zhengzhou, China; 8 Department of Ultrasound, Shanxi Bethune Hospital, Shanxi Academy of Medical Sciences, Taiyuan, China; 9 Department of Medical Ultrasound, Tongji Hospital, Tongji Medical College of Huazhong University of Science and Technology, Wuhan, China; 10 Department of Ultrasound, China-Japan Union Hospital of Jilin University, Changchun, China; 11 Department of Ultrasound, The Sun Yat-Sen Memorial Hospital, Sun Yat-Sen University, Guangzhou, China; 12 Department of Ultrasonography, First Affiliated Hospital of Guangxi Medical University, Nanning, China; 13 Department of Medical Ultrasound, The Second Affiliated Hospital, School of Medicine, Xi’an Jiaotong University, Xi’an, China; 14 Department of Ultrasound, Union Hospital of Fujian Medical University, Fujian Institute of Ultrasound Medicine, Fuzhou, China; 15 Department of Ultrasound, Ruijin Hospital, Shanghai Jiaotong University, School of Medicine, Shanghai, China; 16 Department of Ultrasonography, Renmin Hospital of Wuhan University, Wuhan, China; 17 Department of Ultrasound, Qilu Hospital, Shandong University, Jinan, 250012 China; 18 Department of Ultrasound, The Third Xiangya Hospital of Central South University, Changsha, China; 19 Department of Ultrasound Medicine, Tongren Hospital, Shanghai Jiao Tong University School of Medicine, Shanghai, China; 20 Department of Ultrasonography, The Affiliated Hospital of Guizhou Medical University, Guiyang, China; 21 Department of Ultrasound, The First Hospital of Shanxi Medical University, Taiyuan, China; 22 Department of Ultrasound, The Second Hospital of Dalian Medical University, Dalian, China; 23 Department of Medical Imaging Advanced Research, Shenzhen Mindray Bio-Medical Electronics Co., Ltd., Shenzhen, China

**Keywords:** Deep learning, Ultrasonography, Breast neoplasms, Diagnosis, Artificial intelligence

## Abstract

**Background:**

Studies on deep learning (DL)-based models in breast ultrasound (US) remain at the early stage due to a lack of large datasets for training and independent test sets for verification. We aimed to develop a DL model for differentiating benign from malignant breast lesions on US using a large multicenter dataset and explore the model’s ability to assist the radiologists.

**Methods:**

A total of 14,043 US images from 5012 women were prospectively collected from 32 hospitals. To develop the DL model, the patients from 30 hospitals were randomly divided into a training cohort (*n* = 4149) and an internal test cohort (*n* = 466). The remaining 2 hospitals (*n* = 397) were used as the external test cohorts (ETC). We compared the model with the prospective Breast Imaging Reporting and Data System assessment and five radiologists. We also explored the model’s ability to assist the radiologists using two different methods.

**Results:**

The model demonstrated excellent diagnostic performance with the ETC, with a high area under the receiver operating characteristic curve (AUC, 0.913), sensitivity (88.84%), specificity (83.77%), and accuracy (86.40%). In the comparison set, the AUC was similar to that of the expert (*p* = 0.5629) and one experienced radiologist (*p* = 0.2112) and significantly higher than that of three inexperienced radiologists (*p* < 0.01). After model assistance, the accuracies and specificities of the radiologists were substantially improved without loss in sensitivities.

**Conclusions:**

The DL model yielded satisfactory predictions in distinguishing benign from malignant breast lesions. The model showed the potential value in improving the diagnosis of breast lesions by radiologists.

**Supplementary Information:**

The online version contains supplementary material available at 10.1186/s13244-022-01259-8.

## Key points

- A DL model was developed using a large multicenter dataset.
- The DL model can discriminate between benign and malignant lesions on US effectively.
- The model can improve the performance of radiologists in diagnosing breast cancer.

## Introduction

In China, approximately 303,600 new cases of breast cancer and 70,400 breast cancer-related deaths occurred in 2015, and the incidence and mortality of this disease continue to increase [1–3]. There is no sign that this trend will stop by 2030, particularly in rural areas [3]. The goal of early diagnosing breast cancer is to reduce mortality and provide patients with the most appropriate and least aggressive treatment options [4]. As a widely used screening modality, breast cancer screening with mammography has been demonstrated to reduce breast cancer mortality [5, 6]. However, mammography-based breast cancer screening is not very practical in China [7], because Chinese women tend to have small and dense breasts, and because the peak age of breast cancer onset is younger than that in Western women, both of which are known to affect the diagnostic accuracy and effectiveness of mammography [4]. Ultrasound (US) as a powerful complement to mammography, had a higher sensitivity and diagnostic accuracy [7].

US has become an indispensable tool for the screening and diagnosis of breast cancer due to its safety, lower cost, and ease of accessibility in China [8]. With the introduction of the Breast Imaging Reporting and Data System (BI-RADS) for US, the terminology and criteria for describing and classifying breast lesions have become standardized with good diagnostic performance. Radiologists make diagnoses based on certain macroscopic features on US images (such as shape, margin, and echogenicity), and the breast lesions detected by US can be classified according to the possibility of malignancy of BI-RADS, from benign to high suspicion of malignancy. However, there is a substantial overlap in the sonographic features for benign and malignant lesions [9, 10]. And such assessments often rely on the radiologists’ education and experience and can thus sometimes be subjective and time-consuming. Moreover, a major limitation of US is its low specificity (high false positive rate) in the differentiation of benign from malignant breast lesions [11, 12], which might cause many patients with benign lesions to undergo excessive treatment such as unnecessary biopsy and even aggressive surgery. Therefore, although the benign tumors would not have threatened the health of the patients, they would suffer adverse reactions as a result of treatment [13].

Recently, deep learning (DL) algorithms have become the focus of interest and attention from researchers due to their impressive performance in image recognition tasks [14]. DL models can automatically and quantitatively evaluate complex medical image features with high diagnostic accuracy and efficiency; for example, DL with convolutional neural networks (CNNs) has been widely applied to the pattern recognition and classification of medical images [15]. DL techniques have also achieved comparable performance to human experts in different areas [16, 17] and have the potential to assist doctors with limited experience in improving their performance in disease diagnosis in clinical practice [17, 18]. In the field of breast US, DL has been applied to segment images [19], identify and diagnose breast lesions [20–22], predict axillary lymph node status [23, 24], assess breast cancer molecular subtypes [25, 26], and evaluate the pathological complete response in breast cancer [27]. The diagnosis of breast lesions based on DL thus remains an important and popular research area. Several studies have reported the outstanding diagnostic performance of CNNs for breast cancer diagnosis. However, most of the studies used only training and validation sets from one institution without an independent external test set [28, 29]. Due to the differences in the disease spectrum in different centers, the applicability of trained DL models needs to be further investigated. Furthermore, the datasets utilized in several studies were small [30–32], the patients’ medical information and the pathological type of breast lesions were unknown [22, 29], and the ability of the model to assist radiologists was not reported [22, 28, 31]. Therefore, we aimed to develop a DL model using a large dataset from multiple centers and evaluate its diagnostic performance in the differentiation of benign from malignant breast tumors on US images. Moreover, the performance of the model was compared with that of human radiologists and the ability of the model to assist radiologists was explored.

## Materials and methods

### Patients

A multicenter prospective study was conducted at 32 separate tertiary-care hospitals in China between December 2018 and December 2020. This study was approved by the institutional ethics committee of the principal investigator’s hospital (Peking Union Medical College Hospital) and was registered at ClinicalTrials.gov (ChiCTR1900023916). Written informed consent was obtained before collecting the sonographic images from each patient.

We prospectively recruited patients with pathologically confirmed breast lesions who underwent a US examination. The inclusion criteria were as follows: (1) breast lesions visible on the grayscale US; (2) US examination performed before biopsy or preoperative treatment; and (3) pathological results obtained through needle biopsy or surgical excisional biopsy. For patients with multiple lesions, only the largest or most suspicious lesion on grayscale US was included. Clinical data and information on breast cancer-related risk factors were collected via a face-to-face interview or the patient's electronic medical record, including patient age, body mass index, reproductive history (age at menarche, age at the first live birth, if applicable), history of benign breast disease, history of breast cancer in first-degree relatives, and clinical symptoms (palpable mass, pain, nipple discharge, skin changes). The golden standard was the pathology diagnosis, which was obtained from pathological reports.

### Ultrasound equipment

Ultrasound systems Resona7, Resona7s, Resona7T, Resona8, Resona8T, and DC-80 (Shenzhen Mindray BioMedical Electronics, Co., Ltd., Shenzhen, China) equipped with high-frequency linear transducer probes (L14-5, L11-3, L12-3, or L9-3) were used for the US examinations.

### Image acquisition and interpretation

A unified image acquisition protocol was adopted (Additional file 1: Figure S1). Lesions were viewed in at least two perpendicular planes (longitudinal and transverse) with and without caliper measurements. Other sections which may show suspicious malignant characteristics of the lesion on grayscale US were acquired selectively. The maximum diameter of the lesion was recorded. Sonographic features were described according to the BI-RADS lexicon. Appropriate depth, gain, and focal zone settings were optimized for acquiring high-quality US images. Color Doppler US and strain elastography were available to assist the radiologists in making a better diagnosis. At the same time, the status of axillary lymph nodes was evaluated. Static images of the lesions were saved by the radiologists during real-time scanning and then uploaded onto a website (www.nuqcc.cn) in JPG format. All sonography examinations were performed by radiologists with more than 3 years of experience in performing and interpreting breast US examinations. Prospective BI-RADS US assessment categories were assigned by the radiologists according to the BI-RADS criteria: category 2, benign; category 3, probably benign; category 4 (from 4a to 4c), suspicious for malignancy; and category 5, highly suggestive of malignancy.

### Dataset

**Fig. 1:**
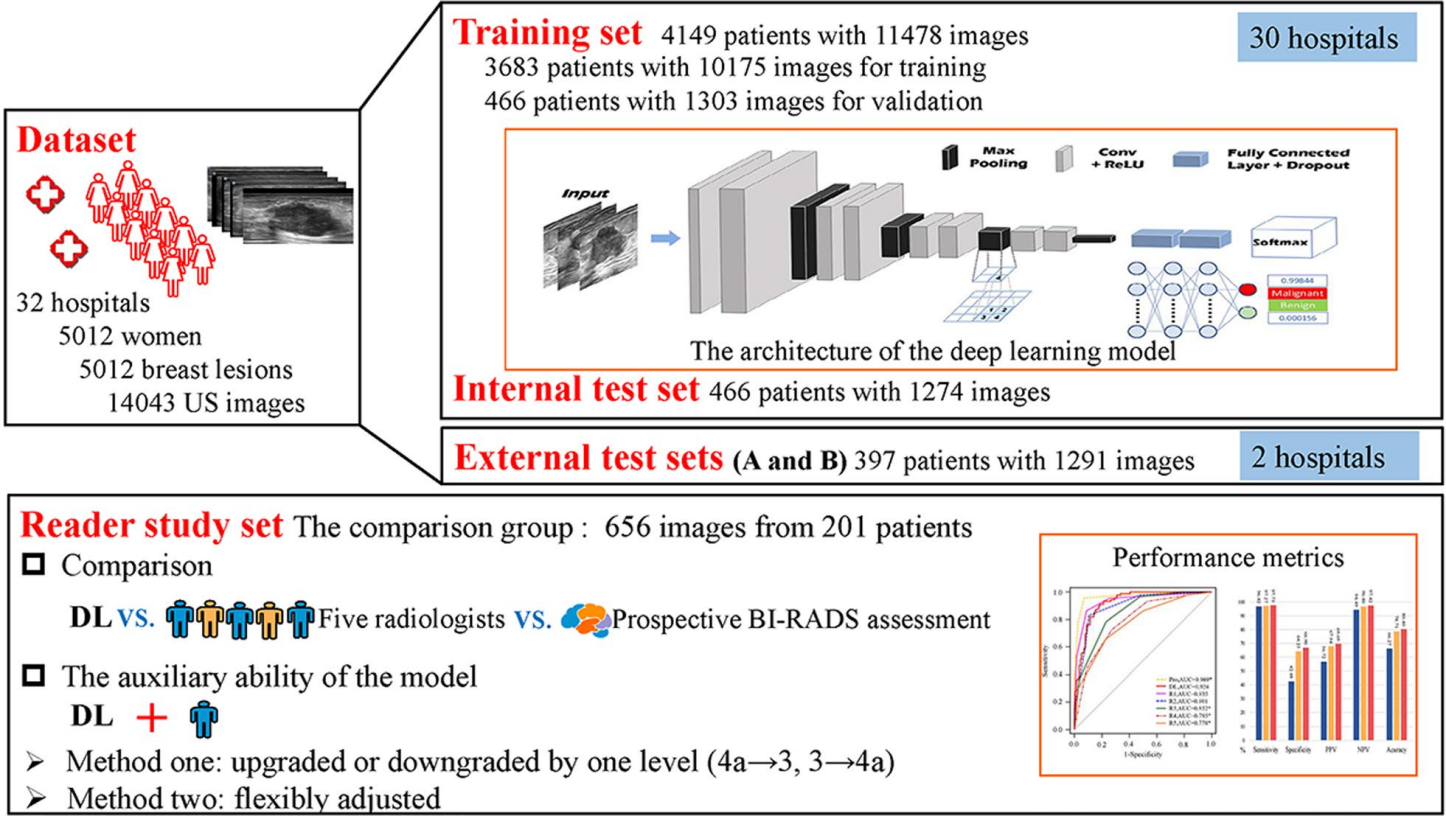
Study workflow. *US* Ultrasound; *Conv* Convolutional Layer; *ReLU* Rectified Linear Unit; *DL* deep learning; *BI-RADS* Breast Imaging Reporting and Data System

B-mode images without caliper measurements were chosen for the DL modeling. Data cleaning was applied to those data and incorrect collections of planes such as mis-uploaded images were discarded. A total of 14,043 qualified B-mode US images from 5012 women were used in this study (Fig. 1). The data from 30 hospitals included 12,752 B-mode US images from 4615 patients were used for the training, validating, and internal testing. The training set contained 4149 patients with 11,478 images and the internal test set included 466 patients with 1274 images. Of the training set, 466 patients (305 benign lesions and 161 malignant lesions) with 1303 images were used for validation, while the remaining 3683 patients (2419 benign lesions and 1264 malignant lesions) with 10,175 images were used to train the model. Two external test sets included 1291 B-mode US images from 397 patients from the remaining 2 hospitals (external test A hospital: 197 patients—97 benign lesions and 100 malignant lesions; external test B hospital: 200 patients—94 benign lesions and 106 malignant lesions) were used to examine the robustness of the model. All the available lesions were used for training and testing the DL model. The internal and external test sets were used to assess the generalizability and robustness of the model. The study workflow is shown in Fig. 1. In addition, we also evaluated our DL model on a publicly available dataset (see Additional file 1: ‘Breast Ultrasound Images Dataset’).

### Deep learning

The assessment of breast lesions in B-mode images, especially for suspicious malignant masses, can be complicated as the morphology of the mass is various and the features of benign and malignant breast lesions can overlap [9, 10]. To acquire a decent performance, we chose the visual geometry group (VGG) deep CNN [33] since we observed that it does not include a global average pooling layer after the last feature extraction layer but directly flattens the feature maps and connects them with three fully connected layers sequentially, which may lead to better diagnostic performance as all extracted significant features are weighted. Indeed, VGG has demonstrated convincing classification accuracy in various medical image analysis tasks [17, 34–37], including breast mass classification [32, 38, 39]. The VGG-19 architecture was adopted as the backbone for breast cancer diagnosis modeling in this study. The detailed architecture is presented in Fig. 1. Dropout layers with a rate of 0.75 were introduced after each dense layer to avoid overfitting, and the last dense layer was changed to have two output neurons with a softmax activation layer to translate the output values into the probabilities of two categories of breast lesions. The category with the highest probability was considered as the result of dichotomies. Pretrained weights on ImageNet were employed for initiating the training in the initial epoch instead of the common He-norm and Xavier initiation to accelerate the convergence.

All B-mode US images were converted to grayscale before preprocessing to eliminate redundant image channels. The masses were labeled using bounding boxes and the annotations were confirmed by an experienced radiologist with 11 years of experience in US. Then, the images were cropped to in which only the lesion area was retained and used as the input to the DL model to reduce the effect of less significant background information. A 50-pixel border to the lesion region was contained to not only capture some surrounding area adjacent to the lesion, which could provide some effective, relevant information, but also avoid incomplete mass extraction. The cropped mass images were resized and padded to 224 × 224 while maintaining their initial aspect ratios. In clinic, radiologists normally read multiple images before giving a diagnosis. However, weighting the prediction of each section is tricky as the order and number of input images are ambiguous, and the majority voting does not embrace the advantage of the DL. To mimic this scenario with minimum increase of time consumption, we stacked three planes according to their patient serial number into one three-channel image for the model. This method not only enables the multi-plane analysis but also reduces the time cost, and can avoid weighting the indeterminate number of diagnostic predictions. The planes of each patient were randomly sampled in the modeling process. The trained model was tested for 10 times to verify its stability. The results are listed in Additional file 1: Table S7 with the mean value and the standard deviation. In comparison study, the planes were sampled in a fixed sequence according to our data acquisition protocol to simulate a real clinical scenario, corresponding results are reported as the final performance of the model in this paper to keep a consistency. The detail of implementations can be found in Additional file 1: ‘Deep learning’.

### Comparison of the diagnostic performance between the model and radiologists

In total, 201 patients with 656 images were randomly selected as a comparison group for the reader study from the internal and external test sets, including 88 malignant breast lesions and 113 benign breast lesions, to compare the diagnostic performance of the model with that of radiologists. Detailed information can be found in Additional file 1: ‘Comparison of the diagnostic performance between the model and radiologists’. Five radiologists with varying experience levels in diagnosing breast lesions independently reviewed and evaluated the images of breast lesions. The multiple B-mode images acquired for a given lesion were shown together for the radiologists' reading to simulate clinical settings. Radiologist 1 is an expert in breast US imaging and has more than 18 years of experience in the US diagnosis of breast. Radiologist 2 has more than 8 years of experience in the US diagnosis of breast. Radiologists 3, 4, and 5 have less than 3 years of experience with breast US. The radiologists were divided into experienced (radiologists 1, 2) and inexperienced (radiologists 3, 4, 5) groups in terms of their experience in US. They were blinded to information regarding the patients’ pathological results, assessment of other readers, or previous classification results. The assessed BI-RADS categories were reported as 2, 3, 4a, 4b, 4c, or 5 by the radiologists. We compared the diagnostic performance of the model with that of the prospective BI-RADS assessment and then with that of these five radiologists.

To explore the auxiliary ability of the model, two methods were used to compare the performance of the radiologists with and without model assistance. In method one, the BI-RADS classification was upgraded or downgraded by one level when the model classified the breast lesion as malignant or benign (an artificial combination of the radiologist's first assessment with the DL predicted result). In other words, BI-RADS 3 category lesions were upgraded to category 4a when the model classified the breast lesion as malignant, and BI-RADS 4a category lesions were downgraded to category 3 when the model classified the breast lesions as benign. In method two, the BI-RADS classification was flexibly adjusted by the radiologists after obtaining the results of the model. Two weeks after the first evaluation of the images, all radiologists reclassified the same images of breast lesions with the knowledge of the predicted probability generated by the model but not with the feedback on the performance of the model and the first evaluation. In brief, the model's result was shown to the radiologists after the radiologists recorded their own BI-RADS assessment, and then, the adjustment was recorded.

### Data and statistical analysis

Continuous variables are described by the mean ± standard deviation (SD), and categorical variables are presented as numbers and percentages. The Student’s t-test and the Chi-square test were applied to compare continuous and categorical variables, respectively. Analysis was made at lesion level. A malignant lesion classified as BI-RADS 4a or higher was considered a true positive, and a benign lesion classified as BI-RADS 2 or 3 was considered a true negative. The trained DL model output the predicted probability scores (continuous 0 to 1) of the malignant and benign lesions based on US images and we selected the class with the highest probability as the binary prediction result (benign or malignant). For example, if the probability value of malignancy was greater than the probability value of benign lesion, we classified the prediction as malignant. That's how we convert the predicted probability value to a binary decision in the upgrade or downgrade calculation (DL model assistance method one) and for the confusion matrix. The sensitivity, specificity, positive predictive value (PPV), negative predictive value (NPV), and accuracy of the model and the radiologists were calculated according to the binary prediction result and the BI-RADS classification, respectively. McNemar’s test was used to compare the sensitivity, specificity, and accuracy, and the Chi-square test was used to compare the PPV and NPV of the model with those of the radiologists. Receiver operating characteristic (ROC) curves were drawn based on the prediction probability derived from the DL model for breast cancer and the BI-RADS category assessment given by radiologists, and the area under the ROC curve (AUC) was obtained. Comparisons between AUCs were performed by using the DeLong test [40]. The F1 score and Matthews correlation coefficient (MCC) were also reported for the test sets. The F1 score represents both the precision and recall of the classification model. The value of the MCC ranges from -1 to 1, and a larger value represents better performance; a value of 1 indicates that the prediction is completely consistent with the actual result. The number of true positive, false positive, false negative, and true negative findings from the model with the test sets were described in a 2 × 2 contingency table representing the confusion matrix. Other statistical methods used can be found in Additional file 1: ‘Data and statistical analysis’.

## Results

### Patients

We included a total of 14,043 US images from 5012 women with breast lesions who were classified prospectively by well-trained radiologists according to BI-RADS. US images from 4615 patients from 30 hospitals were collected and divided into a training set (*n* = 4149; age: 43.67 ± 13.14 years) and an internal test set (*n* = 466; age: 43.21 ± 13.65 years). The 397 patients (age: 44.91 ± 12.06 years) from the other two hospitals were utilized as two independent multicenter test sets. The mean lesion size was 1.84 ± 0.99 cm for the training dataset, 1.76 ± 0.94 cm for the internal test set, and 2.05 ± 1.08 cm for the external test sets. Detailed patient demographics and breast lesion characteristics in the training and test sets are provided in Table 1. The main pathological types of the breast lesions are given in Additional file 1: Table S1.

**Table 1:** Patient demographics and breast lesion characteristics

Characteristic	Training set			Internal test set			External test sets		
	Benign, *n* (%)	Malignant, *n* (%)	*p* value	Benign, *n* (%)	Malignant, *n* (%)	*p* value	Benign, *n* (%)	Malignant, *n* (%)	*p* value
Mean age (range) (y)	43.67 ± 13.14 (10–90)			43.21 ± 13.65 (16–85)			44.91 ± 12.06 (17–80)		
Mean size (range) (cm)	1.84 ± 0.99 (0.30–7.46)			1.76 ± 0.94 (0.40–6.80)			2.05 ± 1.08 (0.35–7.00)		
Patients (*n*)	2724	1425		305	161		191	206	
Images (*n*)	6574	4904		728	546		489	802	
Age (y)	38.99 ± 11.31	52.62 ± 11.70	0.000	38.78 ± 11.94	51.60 ± 12.75	0.000	39.07 ± 10.45	50.32 ± 10.89	0.000
< 30	609 (22.36)	24 (1.68)		77 (25.24)	3 (1.86)		38 (19.90)	1 (0.49)	
30–39	824 (30.25)	162 (11.37)		95 (31.15)	25 (15.53)		63 (32.98)	28 (13.59)	
≥ 40	1291 (47.39)	1239 (86.95)		133 (43.61)	133 (82.61)		90 (47.12)	177 (85.92)	
Age at menarche (y)	13.52 ± 1.50	14.32 ± 1.71	0.000	13.33 ± 1.67	14.32 ± 1.84	0.000	13.85 ± 1.61	14.21 ± 1.78	0.036
Age at first live childbirth (y)	25.21 ± 3.16	25.03 ± 3.51	0.044	25.38 ± 3.50	24.70 ± 3.39	0.056	26.27 ± 3.56	26.00 ± 3.00	0.457
BMI (kg/m^2^)	22.01 ± 3.05	23.64 ± 3.19	0.000	21.73 ± 2.95	23.21 ± 2.92	0.000	22.76 ± 3.32	24.81 ± 3.50	0.000
Maximum diameter (cm)	1.58 ± 0.87	2.34 ± 1.03	0.000	1.53 ± 0.79	2.19 ± 1.05	0.000	1.81 ± 1.03	2.26 ± 1.07	0.000
≤ 2 cm	2107 (77.35)	624 (43.79)		241 (79.02)	82 (50.93)		132 (69.11)	97 (47.09)	
> 2 cm, ≤ 5 cm	597 (21.92)	775 (54.39)		62 (20.33)	76 (47.21)		58 (30.37)	105 (50.97)	
> 5 cm	20 (0.73)	26 (1.82)		2 (0.65)	3 (1.86)		1 (0.52)	4 (1.94)	
Distance from nipple (cm)	2.48 ± 1.49	2.67 ± 1.57	0.000	2.44 ± 1.42	2.56 ± 1.52	0.364	2.63 ± 1.70	3.10 ± 1.69	0.006
Family history (first-degree relatives)		0.943			0.514			0.345	
No	2660 (97.65)	1385 (97.19)		294 (96.39)	157 (97.52)		186 (94.41)	197 (94.98)	
Yes	64 (2.35)	40 (2.81)		11 (3.61)	4 (2.48)		5 (5.59)	9 (5.02)	
History of benign breast disease		0.000			0.045			0.005	
No	2552 (93.69)	1373 (96.35)		284 (93.11)	157 (97.52)		171 (89.53)	199 (96.60)	
Yes	172 (6.31)	52 (3.65)		21 (6.89)	4 (2.48)		20 (10.47)	7 (3.40)	
Clinical symptom(s)			0.000			0.008			0.004
No	675 (24.78)	136 (9.54)		76 (24.92)	23 (14.29)		78 (40.84)	56 (27.18)	
Yes	2049 (75.22)	1289 (90.46)		229 (75.08)	138 (85.71)		113 (59.16)	150 (72.82)	
Position			0.130			0.486			0.513
Left	1390 (50.03)	760 (53.33)		160 (52.46)	79 (49.07)		92 (48.17)	106 (51.46)	
Right	1334 (48.97)	665 (46.67)		145 (47.54)	82 (50.93)		99 (51.83)	100 (48.54)	

Data in parentheses are percentages

*BMI* body mass index

### Performance of the deep learning model

**Fig. 2:**
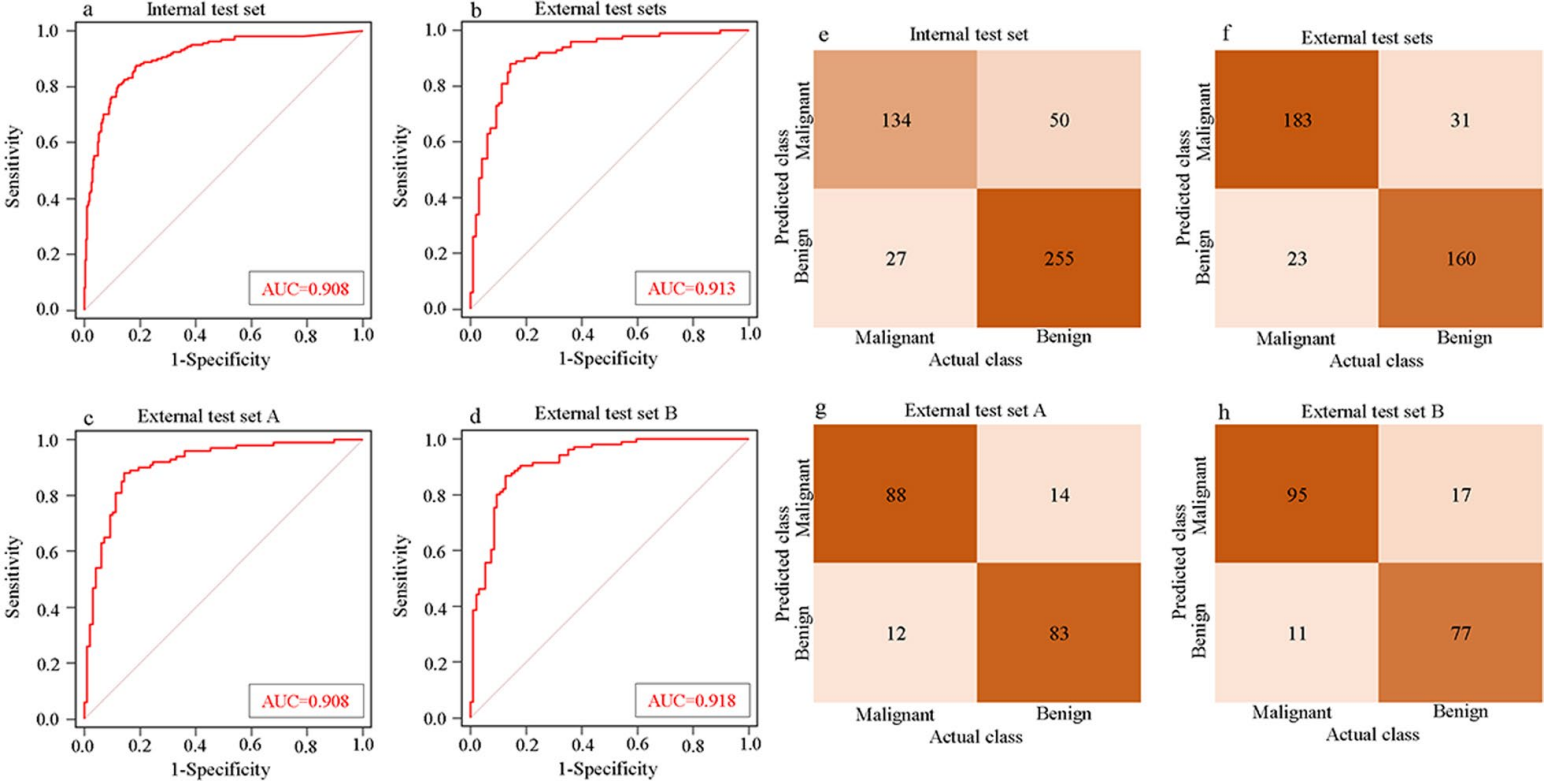
Areas under the receiver operating characteristic curves (AUCs) of the model with the internal test set (**a**), the external test sets (**b**), the external test set A (**c**), and the external test set B (**d**). The confusion matrices of the model in distinguishing benign and malignant breast lesions with the internal test set (**e**), the external test sets (**f**), the external test set A (**g**), and the external test set B (**h**). Actual class, the pathology diagnosis; Predicted class, the binary prediction result of the deep learning model

The DL model achieved a high performance (AUC = 0.908, 95% confidence interval (CI): 0.879–0.933) in distinguishing benign from malignant breast lesions when applied to the breast US images of the internal test set (Fig. 2). The model achieved a lesion-level accuracy of 83.48%, a sensitivity of 83.23%, a specificity of 83.61%, a PPV of 72.83%, and an NPV of 90.43% (Table 2). The effectiveness of the model was evaluated by external test sets with US images obtained from the other two hospitals, with an AUC of 0.913 (95% CI 0.881–0.939), an accuracy of 86.40%, a sensitivity of 88.84%, a specificity of 83.77%, a PPV of 85.51%, and an NPV of 87.43%. For external test set A and B, the AUCs were 0.908 (95% CI 0.859–0.945) and 0.918 (95% CI 0.871–0.952), respectively (Fig. 2 and Table 2). The confusion matrices of the model for predicting breast cancer with the internal test set and external test sets are shown in Fig. 2. Additional file 1: Table S2 shows the performance metrics for the DL model versus the prospective BI-RADS assessment in the test sets.

**Table 2:** Performance metrics for the DL model in the test sets

	AUC (95% CI)	Sensitivity (95% CI)	Specificity (95% CI)	PPV (95% CI)	NPV (95% CI)	ACC	F1	MCC
Internal test set	0.908 (0.879–0.933)	83.23 (76.55–88.65)	83.61 (78.97–87.58)	72.83 (67.33–77.71)	90.43 (86.96–93.04)	83.48 (79.79–86.73)	0.777	0.650
External test sets	0.913 (0.881–0.939)	88.84 (83.72–92.79)	83.77 (77.76–88.70)	85.51 (81.00–89.10)	87.43 (82.48–91.13)	86.40 (82.63–89.61)	0.871	0.728
External test set A	0.908 (0.859–0.945)	88.00 (79.98–93.64)	85.57 (76.97–91.88)	86.28 (79.39–91.12)	87.37 (80.17–92.21)	86.80 (81.26–91.19)	0.871	0.736
External test set B	0.918 (0.871–0.952)	89.62 (82.19–94.71)	81.92 (72.63–89.10)	84.82 (78.34–89.62)	87.50 (79.87–92.51)	86.00 (80.41–90.49)	0.872	0.719

*DL* deep learning, *AUC* area under the receiver operating characteristic curve, *PPV* positive predictive value, *NPV* negative predictive value, *ACC* accuracy, *MCC* Matthews correlation coefficient, *CI* confidence interval

### Comparison between deep learning and radiologists

**Fig. 3:**
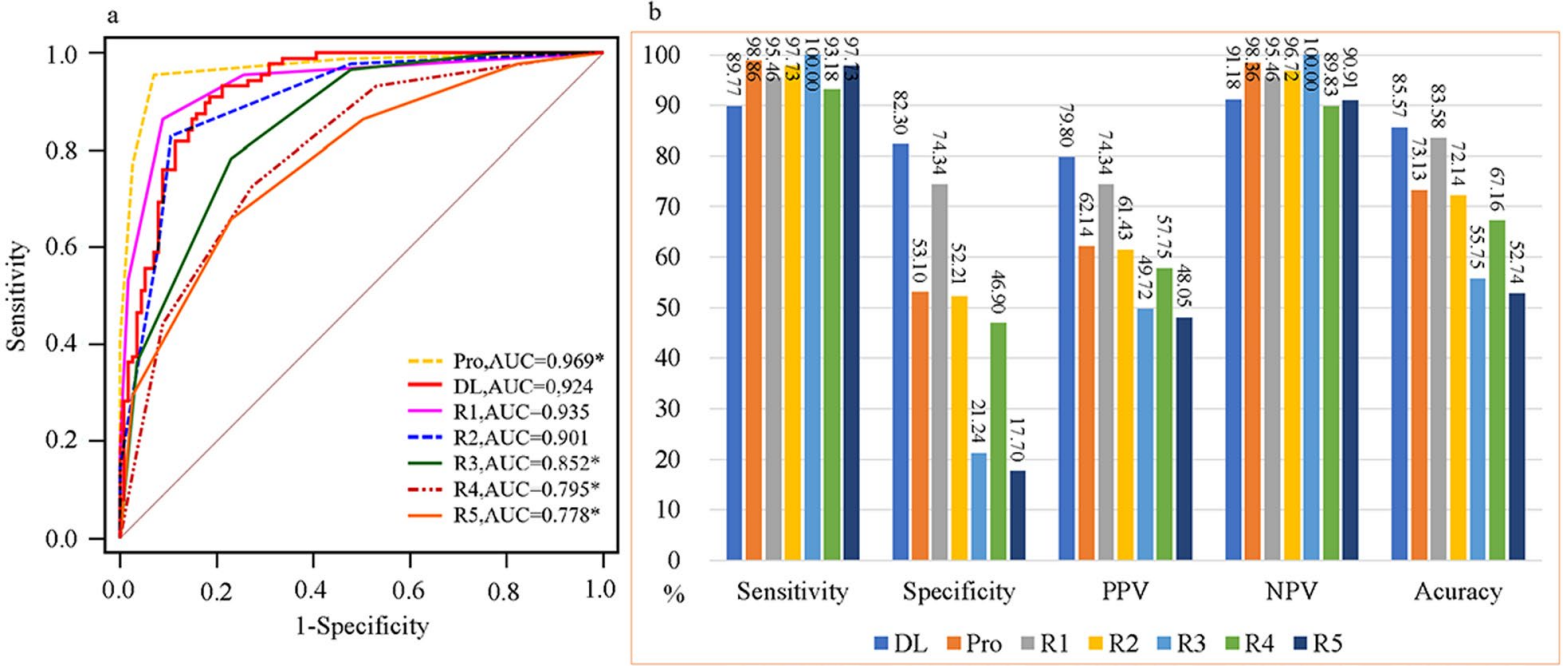
Areas under the receiver operating characteristic curves (AUCs) of the model versus the prospective BI-RADS assessment and the five radiologists in the comparison set (**a**). Performance metrics for the DL model versus the prospective BI-RADS assessment and the five radiologists in the comparison set (**b**). *AUC* area under the receiver operating characteristic curve; *DL* deep learning model; *Pro* the prospective Breast Imaging Reporting and Data System (BI-RADS) assessment; *R* radiologist. *Comparison diagnostic performance with DL model and shows statistical difference

For the comparison analysis, in the comparison set from the internal and external test sets, the results showed that the AUC of the model was lower than that of the prospective BI-RADS assessment (0.969, *p* = 0.0058), and was similar to that of the expert (0.935, *p* = 0.5629) and one experienced radiologist (0.901, *p* = 0.2112) and significantly higher than that of three inexperienced radiologists (*p* < 0.01). The diagnostic accuracy of the model was similar to that of the expert (83.58%, *p* = 0.5966) and was significantly higher than that of the prospective BI-RADS assessment (73.13%), one experienced (72.14%) and three inexperienced radiologists (*p* < 0.01). The specificity of the model was higher than the expert without statistical difference (*p* = 0.0784) and was remarkably higher than that of the prospective BI-RADS assessment and the remaining radiologists (*p* < 0.01). However, the sensitivity of the model was lower than that of the radiologists (Table 3). The AUCs of the model with respect to the prospective BI-RADS assessment and the radiologists are shown in Fig. 3. The performance metrics for the DL model versus the prospective BI-RADS assessment and the five radiologists in the comparison set are shown in Table 3. Additional file 1: Table S3 and S4 demonstrate the performance metrics for the DL model versus the prospective BI-RADS assessment and the radiologists in the comparison set from the internal and external test sets, respectively. Additional file 1: Figure S3 shows the AUCs of the model versus the prospective BI-RADS assessment and the five radiologists in the comparison set from the internal test and the external test sets, respectively.

**Table 3:** Performance metrics for the DL model versus the prospective BI-RADS assessment and the five radiologists in the comparison set

	AUC (95%CI)	*p* value	Sensitivity (95%CI)	*p* value	Specificity (95%CI)	*p* value	PPV (95%CI)	*p* value	NPV (95%CI)	*p* value	ACC (95%CI)	*p* value
DL	0.924 (0.879–0.957)		89.77 (81.47–95.22)		82.30 (74.00–88.84)		79.80 (72.51–85.54)		91.18 (84.69–95.08)		85.57 (79.94–90.12)	
Pro	0.969 (0.934–0.988)	0.0058*	98.86 (93.83–99.97)	0.0078*	53.10 (43.48–62.55)	< 0.0001*	62.14 (57.40–66.67)	0.0036*	98.36 (89.45–99.77)	0.0652	73.13 (66.45–79.13)	0.0005*
R1	0.935 (0.892–0.965)	0.5629	95.46 (88.77–98.75)	0.2266	74.34 (65.27–82.09)	0.0784	74.34 (67.84–79.91)	0.3478	95.46 (88.90–98.22)	0.2454	83.58 (77.72–88.42)	0.5966
R2	0.901 (0.851–0.939)	0.2112	97.73 (92.03–99.72)	0.0391*	52.21 (42.61–61.70)	< 0.0001*	61.43 (56.71–65.94)	0.0025*	96.72 (88.11–99.16)	0.1734	72.14 (65.40–78.22)	0.0002*
R3	0.852 (0.795–0.898)	0.0021*	100 (95.90–100)	0.0039*	21.24 (14.11–29.93)	< 0.0001*	49.72 (47.33–52.11)	< 0.0001*	100	0.1325	55.72 (48.57–62.71)	< 0.0001*
R4	0.795 (0.733–0.849)	< 0.0001*	93.18 (85.75–97.46)	0.5488	46.90 (37.45–56.52)	< 0.0001*	57.75 (53.25–62.12)	0.0004*	89.83 (79.93–95.15)	0.7778	67.16 (60.21–73.61)	< 0.0001*
R5	0.778 (0.714–0.834)	< 0.0001*	97.73 (92.03–99.72)	0.0391*	17.70 (11.16–26.00)	< 0.0001*	48.05 (45.77–50.33)	< 0.0001*	90.91 (70.60–97.66)	0.9682	52.74 (45.59–59.80)	< 0.0001*

*p* value, comparison diagnostic performance with DL model

*DL* deep learning, *BI-RADS* Breast Imaging Reporting and Data System, *AUC* area under the receiver operating characteristic curve, *PPV* positive predictive value, *NPV* negative predictive value, *ACC* accuracy, *CI* confidence interval, *Pro* prospective BI-RADS assessment, *R* radiologist

*p value shows statistical difference

### Comparison of radiologist performance with and without model assistance

**Fig. 4:**
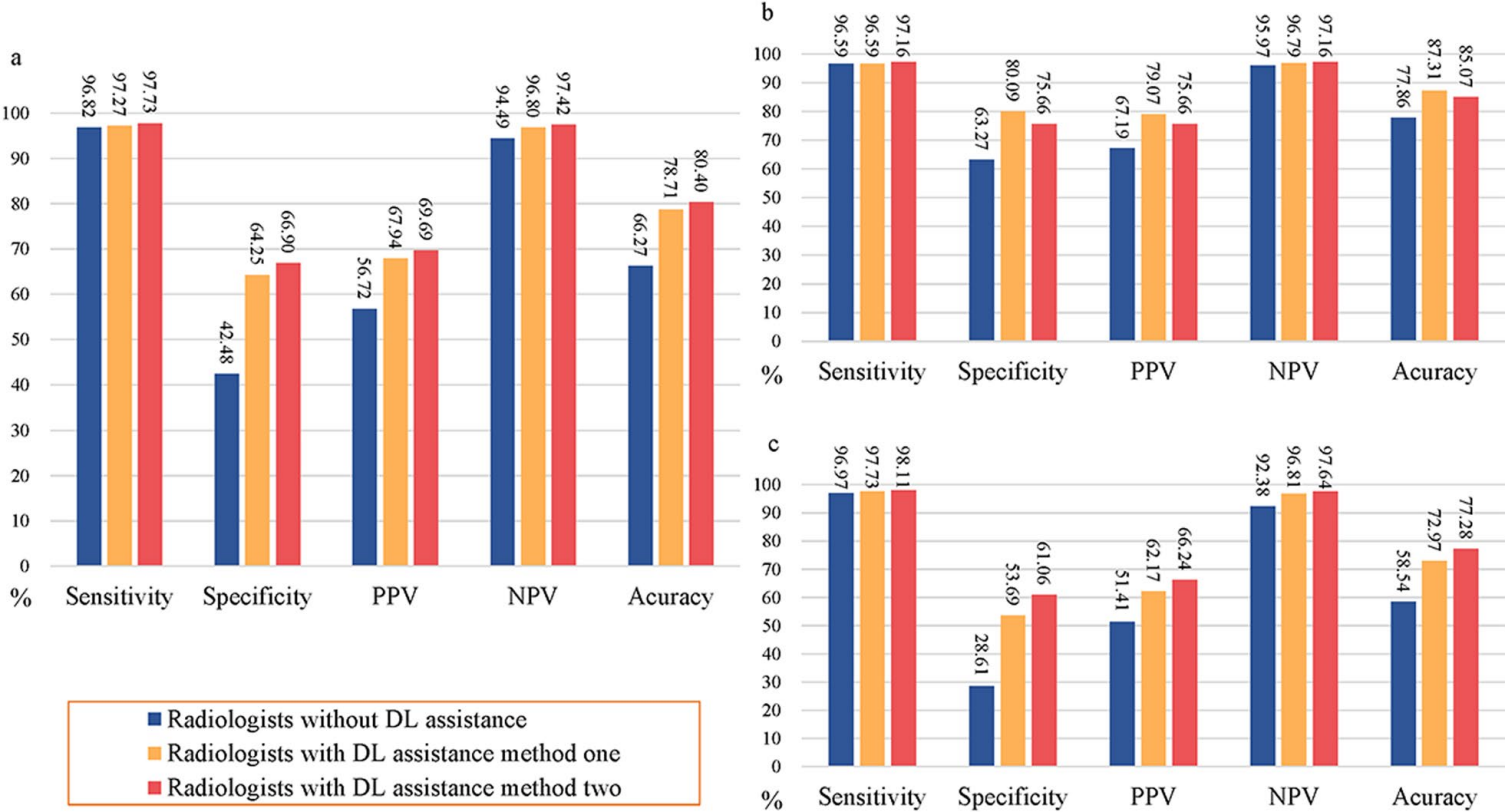
Performance metrics for the radiologists with and without model assistance in the comparison set. **a** All the five radiologists; **b** experienced radiologists; **c** inexperienced radiologists. PPV, positive predictive value; NPV, negative predictive value; DL, deep learning

With the assistance of the model, the average AUC for the diagnostic performance of the experienced radiologists increased but not to a significant degree, while in the inexperienced radiologist group, the average AUC improved significantly. Combined with the prediction of the model, the average accuracies, specificities and PPVs of the experienced and inexperienced radiologists in discriminating benign from malignant lesions on breast US were substantially improved without decrease in sensitivities and NPVs (Table 4). For the experienced radiologists, the average accuracy was considerably improved from 77.86 to 87.31% for method one and 85.07% for method two, and the specificity was markedly improved from 63.27 to 80.09% for method one and 75.66% for method two. For the inexperienced radiologists, the average accuracy was distinctively improved from 58.54 to 72.97% for method one and 77.28% for method two, and the specificity was significantly improved from 28.61 to 53.69% for method one and 61.06% for method two. The diagnostic performance of the model and the radiologists with and without the assistance of the model in discriminating malignant from benign breast lesions are shown in Table 4 and Fig. 4. Detailed information about the diagnostic performance of the five radiologists with the assistance of the model is summarized in Additional file 1: Table S5. The comparison of the diagnostic performance between radiologists with the assistance of method one and method two is summarized in Additional file 1: Table S6. Examples of the DL model in assisting radiologists are shown in Additional file 1: Figure S5.

**Table 4:** Performance metrics for the experienced and inexperienced radiologists with and without model assistance in the comparison set

	AUC (95%CI)	*p* value	Sensitivity (95%CI)	*p* value	Specificity (95%CI)	*p* value	PPV (95%CI)	*p* value	NPV (95%CI)	*p* value	ACC (95%CI)	*p* value
*Radiologists without DL assistance*												
All	0.843 (0.819–0.865)	< 0.0001*	96.82 (94.72–98.25)	< 0.0001*	42.48 (38.36–46.67)	< 0.0001*	56.72 (54.93–58.50)	< 0.0001*	94.49 (91.03–96.66)	0.1065	66.27 (63.25–69.19)	< 0.0001*
Ex	0.919 (0.888–0.944)	0.6778	96.59 (92.73–98.74)	0.0118*	63.27 (56.63–69.57)	< 0.0001*	67.19 (63.27–70.90)	0.0029*	95.97 (91.52–98.14)	0.0774	77.86 (73.48–81.83)	0.0009*
Inex	0.798 (0.764–0.830)	< 0.0001*	96.97 (94.12–98.68)	0.0005*	28.61 (23.86–33.75)	< 0.0001*	51.41 (49.64–53.17)	< 0.0001*	92.38 (85.72–96.08)	0.7031	58.54 (54.49–62.51)	< 0.0001*
*Radiologists with DL assistance method one*												
All	0.861 (0.838–0.881)	< 0.0001^#^	97.27 (95.28–98.58)	0.8036	64.25 (60.14–68.21)	< 0.0001^#^	67.94 (65.46–70.32)	< 0.0001^#^	96.80 (94.52–98.15)	0.1533	78.71 (76.04–81.20)	< 0.0001^#^
Ex	0.932 (0.903–0.954)	0.1044	96.59 (92.73–98.74)	1.0000	80.09 (74.28–85.09)	< 0.0001^#^	79.07 (74.39–83.09)	0.0041^#^	96.79 (93.20–98.52)	0.6885	87.31 (83.66–90.41)	< 0.0001^#^
Inex	0.819 (0.786–0.849)	< 0.0001^#^	97.73 (95.12–99.16)	0.7266	53.69 (48.22–59.09)	< 0.0001^#^	62.17 (59.40–64.86)	0.0011^#^	96.81 (93.18–98.54)	0.0890	72.97 (69.23–76.48)	< 0.0001^#^
*Radiologists with DL assistance method two*												
All	0.908 (0.888–0.925)	< 0.0001^#^	97.73 (95.86–98.91)	0.4545	66.90 (62.85–70.77)	< 0.0001^#^	69.69 (67.14–72.13)	< 0.0001^#^	97.42 (95.33–98.59)	0.0555	80.40 (77.81–82.81)	< 0.0001^#^
Ex	0.933 (0.904–0.955)	0.1202	97.16 (93.49–99.07)	1.0000	75.66 (69.53–81.11)	< 0.0001^#^	75.66 (71.16–79.67)	0.0412^#^	97.16 (93.49–98.79)	0.5564	85.07 (81.21–88.41)	0.0001^#^
Inex	0.902 (0.875–0.924)	< 0.0001^#^	98.11 (95.64–99.38)	0.5488	61.06 (55.65–66.28)	< 0.0001^#^	66.24 (63.17–69.18)	< 0.0001^#^	97.64 (94.54–99.00)	0.0265^#^	77.28 (73.72–80.57)	< 0.0001^#^

*AUC* area under the receiver operating characteristic curve, *PPV* positive predictive value, *NPV* negative predictive value, *ACC* accuracy, *CI* confidence interval, *DL* deep learning, *All* all the five radiologists, *Ex* experienced radiologists, *Inex* inexperienced radiologists

*p values are that of radiologists without DL assistance versus DL and show significant difference

^#^*p* values are that of radiologists with DL assistance vs. radiologists without DL assistance and show significant difference

## Discussion

In this multicenter study, we successfully developed a model for the diagnosis of breast cancer based on sonographic images. The model yielded satisfactory predictions on the test sets, with an AUC of 0.908, a sensitivity of 83.23%, a specificity of 83.61%, and an accuracy of 83.48% for the internal test set and an AUC of 0.913, a sensitivity of 88.84%, a specificity of 83.77%, and an accuracy of 86.40% for the external test sets. With the model assistance, the accuracies and specificities of the radiologists in discriminating benign from malignant lesions on breast US were improved without loss in sensitivities. Our model has potential applicability in the preoperative prediction of breast cancer and shows the potential in helping provide personalized management information for making surgical plans or facilitating pretreatment decisions.

With the continued aging of the population and acceleration in industrialization, urbanization and lifestyle changes in China, the burden of female breast cancer is increasing [1, 41]. As a suitable, convenient and popular examination tool, US has been widely used in breast cancer screening and diagnosis in China. The diagnosis of breast lesions by US mainly relies on the radiologist’s unaided observations to provide an assessment based on the BI-RADS guidelines with high sensitivity at the expense of specificity [11, 42]. In recent years, many new techniques have been developed to compensate for the deficiencies of conventional US [43–45]. In particular, artificial intelligence (AI)- or DL-based computer-aided diagnosis can provide the analyzed results to radiologists as a second opinion or supportive decision and significantly improve the efficiency and effectiveness of the radiologists’ diagnosis [46]. In our study, the diagnostic performance (AUC) of the model was comparable to that of the experienced radiologists and better than that of the inexperienced radiologists in the comparison set. In addition, two methods were used to explore the ability of the model to assist the radiologists, which improved their diagnostic performance to varying degrees. Although the sensitivity of the model was lower than that of the radiologists in the diagnosis of breast cancer, the accuracies and specificities of the radiologists were improved without loss in sensitivities after combining with the prediction of the model. In brief, even the designed three-plane analysis put our model at an inferior position in comparison with radiologists to whom all the planes were shown, it was still capable of recognizing malignant lesions and achieved performance comparable to the assessments of experienced radiologists. It showed the potential value in improving the performance of radiologists in diagnosing breast lesions.

The diagnostic performance of our model was similar to those reported in previous papers on AI methods for breast US analysis [31, 38]. The weaknesses of these studies have been mentioned previously. Two studies using a large dataset of US breast images showed excellent diagnostic performance in the diagnosis of breast cancer. Han et al. [22]developed a GoogLeNet-based model using a large sample of 5151 patients with 7408 US breast images to differentiate between benign and malignant breast lesions, with an AUC over 0.9, an accuracy of approximately 0.90, a sensitivity of 0.86 and a specificity of 0.96. Qi et al. [29] constructed a large dataset containing 8145 breast US images from 2047 patients and developed an automated method for differentiating nonmalignant from malignant breast lesions. Their proposed Mt-Net (BASIC) achieved an accuracy of 93.52%, a specificity of 96.66% and a sensitivity of 87.39% on the test set. However, independent datasets for further assessing the generalizability of the models were lacking. AI computer vision technologies rely on high-quality and large amounts of image data. The quality, quantity and diversity of image data are key factors affecting the accuracy of AI image analysis. Studies on DL in breast US remain at an early stage due to a lack of large datasets for training the models. Multicenter collaborative efforts to obtain data on more types of diseases are conducive to developing a robust, predictive DL model [46]. Studies with independent testing sets are more valuable than studies that depend on internal tests or use only the data from a single center for validation [47]. The assessment of generalizability using an independent dataset is important. In this study, a large-scale dataset containing 14,043 US breast lesion images from 5012 women was prospectively obtained using standardized image acquisition protocols in 32 institutions. Pathological ground truths were obtained for the breast lesions, covering a variety of breast pathology types. Multicenter datasets were used to develop and validate the model, and two external test sets from 2 different institutions were used to demonstrate its robustness and generalizability. The model achieved high diagnostic performance in the differentiation of benign from malignant breast lesions in test sets, which efficiently and reliably confirmed the adaptability of the model.

There are several limitations in our study. First, the DL model was trained based only on B-mode US images, and the diagnostic performance could be improved by including clinical and patient information (such as patient age, size of the lesion, and BI-RADS category assessment) [48], elastography [49], molecular information [23], or other information compared with B-mode US images alone [50]. Second, the performance of the five radiologists who retrospectively reviewed and assessed the images does not necessarily reflect their performance in practice, despite our efforts to simulate real clinical readings. Therefore, the prospective BI-RADS assessment, which was made by radiologists in real time during face-to-face examinations, where they received more information about the patients, was used to compare the diagnostic performance with the model to compensate for this limitation in this study. Additionally, although we strived to acquire a rich and well-generalized dataset in this study, using images collected from systems of a single company could still be a limitation. The radiologists of each hospital participated in the data collection operated with six different devices from one company. The DL model could work better with various data collected from different devices of multiple vendors, and it is a valuable future work to be carried out. In future work, we will develop CNN models at the patient level integrated with more clinical information or elastography and molecular information to build more robust models with better generalizability. It is expected that a real-time AI-based decision support software to be developed and integrated into US machines to help radiologists, especially those with less experience in primary hospitals, to make better diagnoses in clinical practice. Prospective multicenter studies are also required to evaluate the practical application of AI in a real clinical environment.

In summary, we developed a deep learning model using a large dataset that could diagnose breast lesions on US images. The model can provide more objective diagnostic results and improve the performance in diagnosing breast lesions for radiologists. Our study holds great promise for application in the real world to assist radiologists in solving clinical questions.

## Supplementary Information

**Additional file 1.** Supplementary materials, figures and tables.

## Data Availability

The datasets used and/or analyzed during the current study are available from the corresponding author on reasonable request.

## Abbreviations

AI: Artificial intelligence
AUC: Area under the receiver operating characteristic curve
BI-RADS: Breast Imaging Reporting and Data System
CI: Confidence interval
CNN: Convolutional neural network
DL: Deep learning
MCC: Matthews correlation coefficient
NPV: Negative predictive value
PPV: Positive predictive value
US: Ultrasound

## References

[1] Chen W, Zheng R, Baade PD (2016). Cancer statistics in China, 2015. CA Cancer J Clin.

[2] Pan R, Zhu M, Yu C (2017). Cancer incidence and mortality: A cohort study in China, 2008–2013. Int J Cancer.

[3] Lei S, Zheng R, Zhang S (2021). Breast cancer incidence and mortality in women in China: temporal trends and projections to 2030. Cancer Biol Med.

[4] Mainiero MB, Moy L, Expert Panel on Breast I (2017). ACR Appropriateness Criteria((R)) Breast Cancer Screening. J Am Coll Radiol.

[5] Swedish Organised Service Screening Evaluation G (2006). Reduction in breast cancer mortality from organized service screening with mammography: 1. Further confirmation with extended data. Cancer Epidemiol Biomark Prev.

[6] Marmot MG, Altman DG, Cameron DA, Dewar JA, Thompson SG, Wilcox M (2013). The benefits and harms of breast cancer screening: an independent review. Br J Cancer.

[7] Shen S, Zhou Y, Xu Y (2015). A multi-centre randomised trial comparing ultrasound vs mammography for screening breast cancer in high-risk Chinese women. Br J Cancer.

[8] Jiang T, Jiang Y, Chen W (2018). Chinese association of ultrasound in medicine and engineering, superficial organs and peripheral vessels committee expert consensus on clinical frequently asked questions in breast ultrasonography. J Cancer Res Ther.

[9] Zonderland HM, Coerkamp EG, Hermans J, van de Vijver MJ, van Voorthuisen AE (1999). Diagnosis of breast cancer: contribution of US as an adjunct to mammography. Radiology.

[10] Jackson VP (1995). The current role of ultrasonography in breast imaging. Radiol Clin N Am.

[11] Hooley RJ, Scoutt LM, Philpotts LE (2013). Breast ultrasonography: state of the art. Radiology.

[12] Lee SH, Cho N, Chang JM (2014). Two-view versus single-view shear-wave elastography: comparison of observer performance in differentiating benign from malignant breast masses. Radiology.

[13] Siu AL, Force USPST (2016). Screening for breast cancer: U.S. Preventive Services Task Force recommendation statement. Ann Intern Med.

[14] LeCun Y, Bengio Y, Hinton G (2015). Deep learning. Nature.

[15] Litjens G, Kooi T, Bejnordi BE (2017). A survey on deep learning in medical image analysis. Med Image Anal.

[16] Zhou W, Yang Y, Yu C (2021). Ensembled deep learning model outperforms human experts in diagnosing biliary atresia from sonographic gallbladder images. Nat Commun.

[17] Hu H, Gong L, Dong D (2021). Identifying early gastric cancer under magnifying narrow-band images with deep learning: a multicenter study. Gastrointest Endosc.

[18] Yang Q, Wei J, Hao X (2020). Improving B-mode ultrasound diagnostic performance for focal liver lesions using deep learning: a multicentre study. EBioMedicine.

[19] Hu Y, Guo Y, Wang Y (2019). Automatic tumor segmentation in breast ultrasound images using a dilated fully convolutional network combined with an active contour model. Med Phys.

[20] Yap MH, Pons G, Marti J (2018). Automated breast ultrasound lesions detection using convolutional neural networks. IEEE J Biomed Health Inform.

[21] Cao Z, Duan L, Yang G, Yue T, Chen Q (2019). An experimental study on breast lesion detection and classification from ultrasound images using deep learning architectures. BMC Med Imaging.

[22] Han S, Kang HK, Jeong JY (2017). A deep learning framework for supporting the classification of breast lesions in ultrasound images. Phys Med Biol.

[23] Zheng X, Yao Z, Huang Y (2020). Deep learning radiomics can predict axillary lymph node status in early-stage breast cancer. Nat Commun.

[24] Zhou LQ, Wu XL, Huang SY (2020). Lymph node metastasis prediction from primary breast cancer US images using deep learning. Radiology.

[25] Jiang M, Zhang D, Tang SC (2021). Deep learning with convolutional neural network in the assessment of breast cancer molecular subtypes based on US images: a multicenter retrospective study. Eur Radiol.

[26] Zhang X, Li H, Wang C (2021). Evaluating the accuracy of breast cancer and molecular subtype diagnosis by ultrasound image deep learning model. Front Oncol.

[27] Jiang M, Li CL, Luo XM (2021). Ultrasound-based deep learning radiomics in the assessment of pathological complete response to neoadjuvant chemotherapy in locally advanced breast cancer. Eur J Cancer.

[28] Zhang H, Han L, Chen K, Peng Y, Lin J (2020). Diagnostic efficiency of the breast ultrasound computer-aided prediction model based on convolutional neural network in breast cancer. J Digit Imaging.

[29] Qi X, Zhang L, Chen Y (2019). Automated diagnosis of breast ultrasonography images using deep neural networks. Med Image Anal.

[30] Xiao T, Liu L, Li K, Qin W, Yu S, Li Z (2018). Comparison of transferred deep neural networks in ultrasonic breast masses discrimination. Biomed Res Int.

[31] Wan KW, Wong CH, Ip HF (2021). Evaluation of the performance of traditional machine learning algorithms, convolutional neural network and AutoML Vision in ultrasound breast lesions classification: a comparative study. Quant Imaging Med Surg.

[32] Byra M, Galperin M, Ojeda-Fournier H (2019). Breast mass classification in sonography with transfer learning using a deep convolutional neural network and color conversion. Med Phys.

[33] Simonyan K, Zisserman A (2014) Very deep convolutional networks for large-scale image recognition. 10.48550/arXiv.1409.1556

[34] Boumaraf S, Liu X, Wan Y (2021). Conventional machine learning versus deep learning for magnification dependent histopathological breast cancer image classification: a comparative study with visual explanation. Diagnostics.

[35] Zhu J, Zhang S, Yu R (2021). An efficient deep convolutional neural network model for visual localization and automatic diagnosis of thyroid nodules on ultrasound images. Quant Imaging Med Surg.

[36] Wang Y, Guan Q, Lao I (2019). Using deep convolutional neural networks for multi-classification of thyroid tumor by histopathology: a large-scale pilot study. Ann Transl Med.

[37] Kim KE, Kim JM, Song JE, Kee C, Han JC, Hyun SH (2020). Development and validation of a deep learning system for diagnosing glaucoma using optical coherence tomography. J Clin Med.

[38] Tanaka H, Chiu SW, Watanabe T, Kaoku S, Yamaguchi T (2019). Computer-aided diagnosis system for breast ultrasound images using deep learning. Phys Med Biol.

[39] Daoud MI, Abdel-Rahman S, Bdair TM, Al-Najar MS, Al-Hawari FH, Alazrai R (2020). Breast tumor classification in ultrasound images using combined deep and handcrafted features. Sensors.

[40] DeLong ER, DeLong DM, Clarke-Pearson DL (1988). Comparing the areas under two or more correlated receiver operating characteristic curves: a nonparametric approach. Biometrics.

[41] DeSantis CE, Ma J, Gaudet MM (2019). Breast cancer statistics, 2019. CA Cancer J Clin.

[42] Yoon JH, Lee HS, Kim YM (2019). Effect of training on ultrasonography (US) BI-RADS features for radiology residents: a multicenter study comparing performances after training. Eur Radiol.

[43] Barr RG, Nakashima K, Amy D (2015). WFUMB guidelines and recommendations for clinical use of ultrasound elastography: part 2: breast. Ultrasound Med Biol.

[44] Menezes GLG, Pijnappel RM, Meeuwis C (2018). Downgrading of breast masses suspicious for cancer by using optoacoustic breast imaging. Radiology.

[45] Guo R, Lu G, Qin B, Fei B (2018). Ultrasound imaging technologies for breast cancer detection and management: a review. Ultrasound Med Biol.

[46] Chan HP, Samala RK, Hadjiiski LM (2020). CAD and AI for breast cancer-recent development and challenges. Br J Radiol.

[47] Kuang M, Hu HT, Li W, Chen SL, Lu XZ (2021). Articles that use artificial intelligence for ultrasound: a reader's guide. Front Oncol.

[48] Liao WX, He P, Hao J (2020). Automatic identification of breast ultrasound image based on supervised block-based region segmentation algorithm and features combination migration deep learning model. IEEE J Biomed Health Inform.

[49] Zhang Q, Xiao Y, Dai W (2016). Deep learning based classification of breast tumors with shear-wave elastography. Ultrasonics.

[50] Yi J, Kang HK, Kwon JH (2021). Technology trends and applications of deep learning in ultrasonography: image quality enhancement, diagnostic support, and improving workflow efficiency. Ultrasonography.

