# Supplementary material for: Deep learning based on ultrasound images assists breast lesion diagnosis in China: a multicenter diagnostic study
Source: Insights Imaging. 2022 Jul 28;13:124. doi: 10.1186/s13244-022-01259-8 (PMC9334487; doi:10.1186/s13244-022-01259-8)
Supplement: Supplementary file 1 — Additional file 1. Supplementary materials, figures and tables. [file 13244_2022_1259_MOESM1_ESM.pdf]

ELECTRONIC SUPPLEMENTARY MATERIAL

Deep learning based on ultrasound images assists breast lesion diagnosis in China: a multicenter diagnostic study

Materials and Methods

Deep learning

Preliminary experiments with several popular state-of-the-art backbones including ResNets [1], EfficientNetsV2 [2], and VGG were conducted for model selection, and the results confirmed that the VGG 19 had the ability to achieve superior performance. ResNet-18, ResNet-50, EfficientNetV2-S, VGG-16, and VGG-19 were all trained with identical training parameters such as learning rate, optimizer, criterion function, epochs, etc. The trained models were tested with the validation set without the random multi-plane sampling. The comparison is shown in Supplementary Table 8. VGG-19 performed better in AUC, accuracy, sensitivity, PPV, and NPV than others. Despite that EfficientV2-S performed better in specificity, its sensitivity was lower, indicating that more malignant lesions would be missed. VGG-19 acquired balanced results with close performance in sensitivity and specificity. Therefore, we chose to use VGG-19 for our study.

Data augmentation was applied in the study. We flipped the data horizontally and randomly applied contrast enhancement at a ratio between 0 and 2 and/or a rotation between -10° and 10°. The experiment was conducted on a 12 GB NVIDIA TITAN V GPU. SGD was utilized as the optimizer and the cosine annealing schedule was applied for scheduling the learning rate from 1×10^{-2} to 1×10^{-6}. The cross-entropy was used as the loss function. The model was trained for 100 epochs, with a batch size of 64. The TensorBoard was used to visualize changes in the loss and accuracy (Supplementary Figure 2), which allowed us to observe the training process. Libraries used for image processing and DL modelling included Python (3.6.7), NumPy (1.19.0), PyTorch (1.1.0), TensorboardX (2.1), PIL (7.2.0), and scikit-learn (0.21.3). The pretrained model weights were downloaded from https://download.pytorch.org/models/vgg19-dcbb9e9d.pth.

Visualization of the model

The Class Activation Mapping (CAM) algorithm [3] was employed to visualize how our model interpreted the breast US images for predicting the risk of breast lesions. This algorithm applies color mapping to images according to the weight of the feature extraction layer of the model, and creates heatmaps in which the color is warmer in areas of strong attention and colder in areas of weak attention (Supplementary Figure 4).

Comparison of the diagnostic performance between the model and radiologists

Among the 201 patients randomly selected from the test sets, 101 patients (39 malignant breast lesions and 62 benign breast lesions) with 307 images were randomly selected from the internal test set, and 100 patients (49 malignant breast lesions and 51 benign breast lesions) with 349 images were randomly selected from the external test sets. The comparison of the diagnostic performance between the model and radiologists in the comparison set from the internal test set and external test sets is shown in Supplementary Table 3 and Supplementary Table 4, respectively.

Data and statistical analysis

The intra-observer variability between the radiologist's assessment in the first reading alone and the assessment in the second reading before showing the DL model prediction was calculated by using kappa statistics. The level of agreement (kappa value, κ) was defined as follows: ≤ 0.20, slight agreement; 0.21–0.40, fair agreement; 0.41–0.60, moderate agreement; 0.61–0.80, substantial agreement; and 0.81–1.00, almost perfect agreement [4-5]. The intra-observer agreement in BI-RADS assessment was perfect for radiologist 1, substantial for radiologist 2, and moderate for radiologists 3,4 and 5 (Supplementary Table 9).

Decision curve analysis (DCA) was used to test the clinical usefulness of the DL model and radiologists with and without model assistance in breast cancer prediction. DCA was performed to evaluate the clinical utility of the prediction model by quantifying the net benefits when different threshold probabilities were considered [6-8]. The y-axis measures the net benefit. The x-axis shows the corresponding risk threshold. The grey line represents the assumption that all lesions were malignant lesions. The black straight line represents the assumption that all lesions were benign lesions (Supplementary Figure 6).

Statistical analyses were performed by using SPSS software (version 25.0 for Windows; IBM Corporation, Armonk, NY), MedCalc Statistical Software (version 18.2.1, MedCalc Software bvba, Ostend, Belgium), and R language (version 3.6.2). A *P* value of <0.05 was considered statistically significant.

Breast Ultrasound Images Dataset

In addition, we also evaluated our DL model on the publicly available Breast Ultrasound Images (BUSI) dataset [9]. The BUSI dataset contains 437 benign images, 210 malignant images, and 133 normal images. Normal images were excluded in the study as while as the images with marks (caliper measurement, vascularity, and text) blocking the mass, and only one mass on each image were included. A total of 504 images (504 masses) were used in this study. The DL model achieved an AUC of AUC=0.864 (95% CI: 0.831-0.893), an accuracy of 80.16% (95% CI: 76.41%-83.55%), a sensitivity of 79.31% (95% CI: 72.53%-85.07%), a specificity of 80.61% (95% CI: 75.92%-84.73%), a PPV of 68.32% (95% CI: 63.08%-73.13%), and an NPV of 88.08% (95% CI: 84.61%-90.85%) in BUSI dataset.

Supplementary References:

1 He KM, Zhang XY, Ren SQ, SJ (2015) Deep Residual Learning for Image Recognition. https://doi.org/10.48550/arXiv.1512.03385
2 Tan MX, Le QV (2021) EfficientNetV2: Smaller Models and Faster Training. https://doi.org/10.48550/arXiv.2104.00298
3 Selvaraju RR, Das A, Vedantam R, Cogswell M, Parikh D, Batra D (2016) Grad-CAM: Visual Explanations from Deep Networks via Gradient-Based Localization. https://doi.org/10.48550/arXiv.1610.02391
4 Landis JR, Koch GG (1977) The measurement of observer agreement for categorical data. Biometrics 33:159-174
5 Viera AJ, Garrett JM (2005) Understanding interobserver agreement: the kappa statistic. Fam Med 37:360-363
6 Vickers AJ, Elkin EB (2006) Decision curve analysis: a novel method for evaluating prediction models. Med Decis Making 26:565-574
7 Kerr KF, Brown MD, Zhu K, Janes H (2016) Assessing the Clinical Impact of Risk Prediction Models With Decision Curves: Guidance for Correct Interpretation and Appropriate Use. J Clin Oncol 34:2534-2540
8 Vickers AJ, Cronin AM, Elkin EB, Gonen M (2008) Extensions to decision curve analysis, a novel method for evaluating diagnostic tests, prediction models and molecular markers. BMC Med Inform Decis Mak 8:53

9 Al-Dhabyani W, Gomaa M, Khaled H, Fahmy A (2020) Dataset of breast ultrasound images. Data Brief 28:104863

Supplementary Figures and Tables

Supplementary Figure 1 Unified image acquisition protocol.

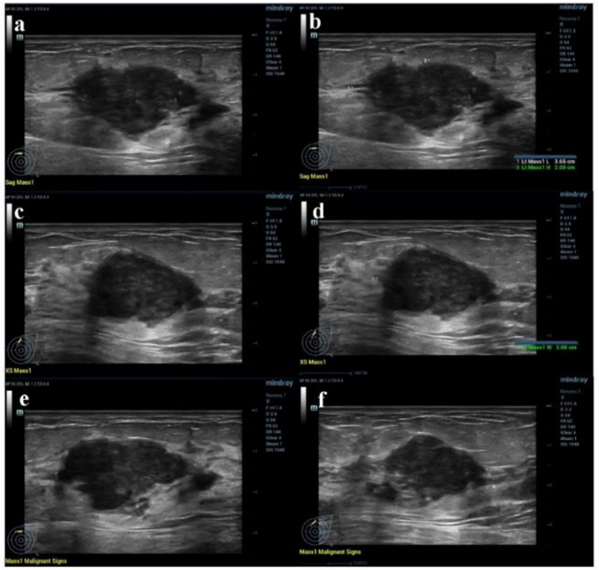

(a, c) Longitudinal and transverse planes without caliper measurements. (b, d) Longitudinal and transverse planes with caliper measurements. (e, f) Grayscale images of other planes.

Supplementary Figure 2 Accuracy and loss curves with the training set and the validation set.

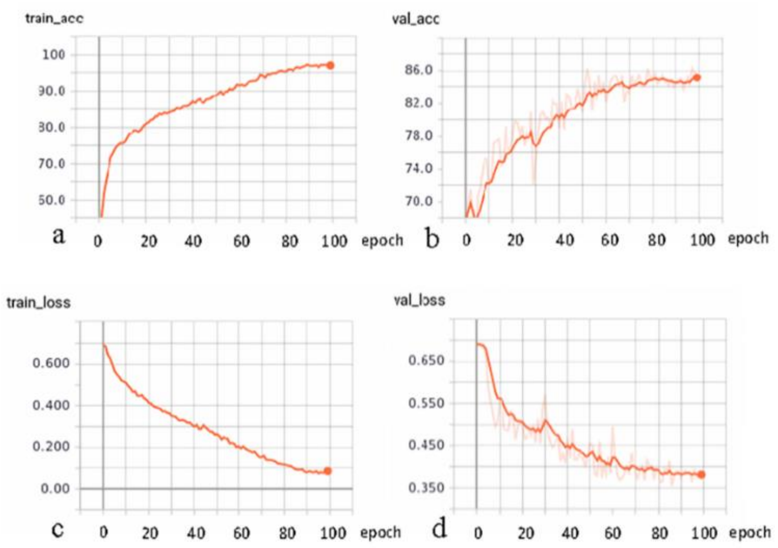

The first row shows the accuracy curves of the training and validation phases (a, b), and the second row shows the loss curves (c, d). Val=validation, acc= accuracy.

Supplementary Figure 3 Areas under the receiver operating characteristic curves (AUCs) of the model versus the prospective BI-RADS assessment and the five radiologists in the comparison set from the internal test (a) and the external test sets(b).

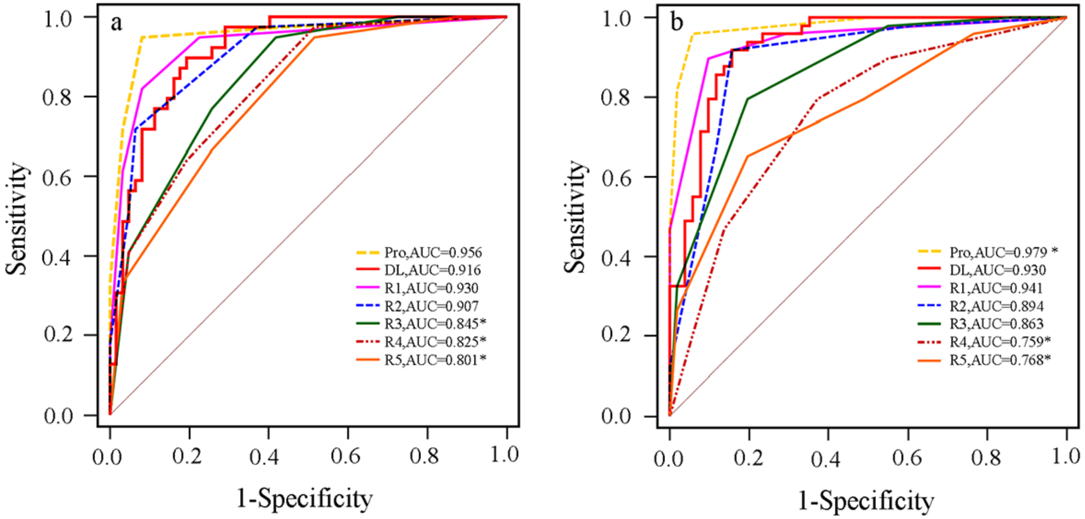

DL=deep learning; AUC= area under the receiver operating characteristic curve; BI-RADS, Breast Imaging Reporting and Data System; Pro=the prospective BI-RADS assessment; R=radiologist. * Comparison diagnostic performance with DL model and shows significant difference.

Supplementary Figure 4 US images and heatmaps of the significant regions of the breast malignant (a) and benign (b) lesions using class activation mapping (CAM).

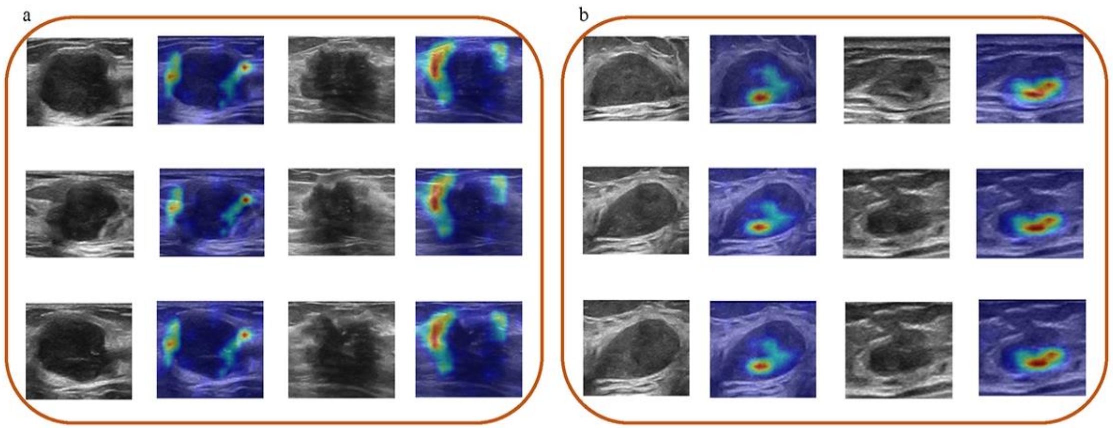

Supplementary Figure 5 Examples of DL model in assisting radiologists.

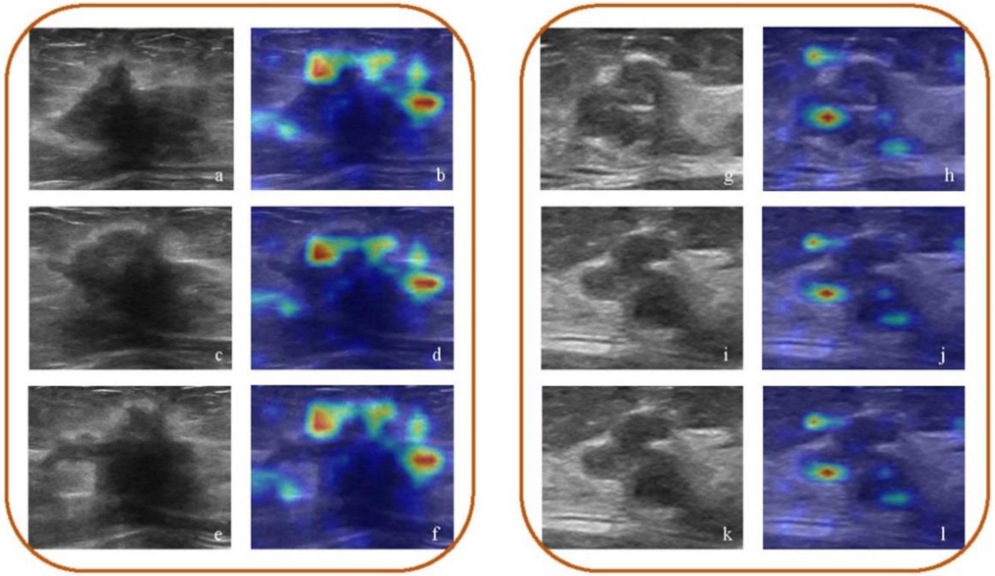

The images of the lesion on B-mode US (a, c, e & g, i, k) and heatmaps (b, d, f & h, j, l). (Left) A 51-year-old woman with palpable breast mass, which was confirmed as invasive ductal carcinoma. This lesion was classified as Breast Imaging-Reporting and Data System (BI-RADS) category 4c, 4c, 5, 5, and 5 by the five radiologists, respectively. The DL model output the predicted probability scores were 0.999 and 0.001 for malignant and benign lesion, respectively. The binary prediction result was malignant. The DL model provided a strong suggestion of malignancy to radiologists as a supportive decision and enhanced the confidence of the radiologists' diagnoses. (Right) A 35-year-old woman with asymptomatic breast mass, which was confirmed as fibroadenoma. This lesion was classified as Breast Imaging-Reporting and Data System (BI-RADS) category 3, 4a, 4a, 4a, and 4a by the five radiologists, respectively. The DL model output the predicted probability scores were 0.016 and 0.984 for malignant and benign lesion, respectively. The binary prediction result was benign. The breast lesion was downgraded to category 3 by using method one for one experienced and three inexperienced radiologists.

Supplementary Figure 6 Comparison of decision curves of breast cancer prediction in the comparison between the DL model and radiologists with and without model assistance in the comparison set.

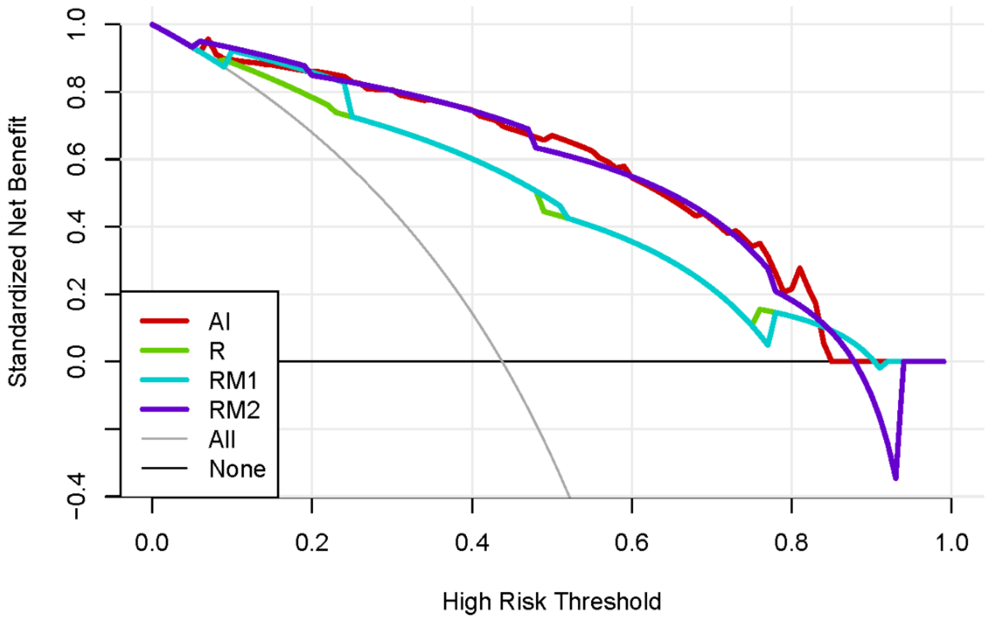

The decision curves indicate that using the DL model (red line) to predict breast cancer adds more benefit to patients than the radiologists alone (green line) in the comparison set. The radiologists with model assistance-method two (purple line) brings more benefit than the radiologists with model assistance-method one (blue line) in the comparison set. AI=artificial intelligence, R= radiologists without model assistance, RM1= radiologists with model assistance method one, RM2= radiologists with model assistance method two.

Supplementary Table 1

Main pathological breast lesion types

Pathological type	Training set, N (%)	Internal test set, N (%)	External test sets, N (%)
Malignant lesions	1425 (34.35)	161 (34.55)	206 (51.89)
Invasive carcinoma	1288 (31.05)	143 (30.70)	171 (43.08)
Ductal carcinoma in situ	110 (2.65)	14 (3.01)	22 (5.54)
Solid papillary carcinoma	10 (0.24)	1 (0.21)	5 (1.26)
Encapsulated papillary carcinoma	6 (0.15)	0 (0.00)	5 (1.26)
Lobular tumor, malignant/borderline	3 (0.07)	1 (0.21)	2 (0.50)
Other malignant lesions [a]	8 (0.19)	2 (0.42)	1 (0.25)
Benign lesions	2724 (65.65)	305 (65.45)	191 (48.11)
Fibrocystic disease and adenosis	660 (15.91)	65 (13.95)	22 (5.54)
Fibroadenoma	1764 (42.52)	203 (43.56)	119 (29.97)
Intraductal papilloma	142 (3.42)	19 (4.08)	27 (6.80)
Lobular tumor, benign	28 (0.67)	1 (0.21)	9 (2.27)
Inflammation	86 (2.07)	10 (2.15)	5 (1.26)
Cyst, benign	13 (0.31)	3 (0.64)	3 (0.76)
Other benign lesions [b]	31 (0.75)	4 (0.86)	6 (1.51)
Total	4149 (100.00)	466 (100.00)	397 (100.00)

Note—Numbers in parentheses are percentages.

[a] Pathological findings included intraductal papillary carcinoma, malignant lymphoma, microscopic invasive carcinoma, and Paget disease.

[b] Pathological findings included atypical ductal hyperplasia, sclerosing adenosis, lipoma, radial scar, hamartoma, adenomyoepithelioma, fat necrosis, nodular fasciitis, and tubular adenoma.

Supplementary Table 2

Performance metrics for the DL model versus the prospective BI-RADS assessment in the test sets

Test sets		AUC (95% CI)	Sensitivity (95% CI)	Specificity (95% CI)	PPV (95% CI)	NPV (95% CI)	ACC	F1	MCC
Internal test set	DL	0.908 (0.879-0.933)	83.23 (76.55-88.65)	83.61 (78.97-87.58)	72.83 (67.33-77.71)	90.43 (86.96-93.04)	83.48 (79.79-86.73)	0.777	0.650
	Pro	0.969 (0.948-0.982)	99.38 (96.59-99.98)	59.67 (53.93-65.22)	56.54 (53.14-59.87)	99.45 (96.26-99.92)	73.39 (69.13-77.35)	0.721	0.575
	P value	<0.0001*	<0.0001*	<0.0001*	0.0004*	0.0001*	<0.0001*		
External test sets	DL	0.913 (0.881-0.939)	88.84 (83.72-92.79)	83.77 (77.76-88.70)	85.51 (81.00-89.10)	87.43 (82.48-91.13)	86.40 (82.63-89.61)	0.871	0.728
	Pro	0.952 (0.926-0.971)	99.52 (97.33-99.99)	47.12 (39.87-54.46)	66.99 (63.96-69.89)	98.90 (92.68-99.84)	74.31 (69.71-78.54)	0.801	0.554
	P value	0.0079*	<0.0001*	<0.0001*	<0.0001*	0.0016*	<0.0001*		
External test set A	DL	0.908 (0.859-0.945)	88.00 (79.98-93.64)	85.57 (76.97-91.88)	86.28 (79.39-91.12)	87.37 (80.17-92.21)	86.80 (81.26-91.19)	0.871	0.736
	Pro	0.949 (0.908-0.975)	99.00 (94.55-99.98)	47.42 (37.19-57.82)	66.00 (61.62-70.13)	97.87 (86.61-99.70)	73.60 (66.89-79.62)	0.792	0.545
	P value	0.0500	0.0010*	<0.0001*	0.0003*	0.0418*	0.0007*		
External test set B	DL	0.918 (0.871-0.952)	89.62 (82.19-94.71)	81.92 (72.63-89.10)	84.82 (78.34-89.62)	87.50 (79.87-92.51)	86.00 (80.41-90.49)	0.872	0.719
	Pro	0.957 (0.919-0.981)	100 (96.58-100)	46.81 (36.44-57.39)	67.95 (63.69-71.93)	100 /	75.00 (68.40-80.84)	0.809	0.564
	P value	0.0533	0.0010*	<0.0001*	0.0017*	0.0147*	0.0026*		

BI-RADS, Breast Imaging Reporting and Data System; DL, deep learning; Pro, prospective BI-RADS assessment; AUC, area under the receiver operating characteristic curve; PPV, positive predictive value; NPV, negative predictive value; ACC, accuracy; MCC, Matthews correlation coefficient; CI, confidence interval.

P value, comparison diagnostic performance with DL model.

[*]*P* value shows statistical difference.

Supplementary Table 3

Performance metrics for the DL model versus the prospective BI-RADS assessment and the five radiologists in the comparison set from the internal test set

	AUC (95%CI)	*P* value	Sensitivity (95%CI)	*P* value	Specificity (95%CI)	*P* value	PPV (95%CI)	*P* value	NPV (95%CI)	*P* value	ACC	*P* value
DL	0.916 (0.844-0.962)		84.62 (69.47-94.14)		83.87 (72.33-91.99)		76.74 (64.81-85.53)		89.66 (80.4-94.80)		84.16 (75.55-90.66)	
Pro	0.956 (0.896-0.987)	0.0959	97.44 (86.52-99.94)	0.0625	56.45 (43.26-69.01)	0.0001*	58.46 (51.35-65.24)	0.0511	97.22 (83.32-99.59)	0.1766	72.28 (62.48-80.72)	0.0227*
R1	0.930 (0.862-0.971)	0.6163	94.87 (82.68-99.37)	0.2188	77.42 (65.03-87.07)	0.3877	72.55 (62.39-80.82)	0.6439	96.00 (86.07-98.94)	0.2115	84.16 (75.55-90.66)	1.0000
R2	0.907 (0.833-0.956)	0.6902	97.44 (86.52-99.94)	0.0625	62.90 (49.69-74.84)	0.0023*	62.30 (54.34-69.64)	0.1208	97.50 (84.81-99.63)	0.1403	76.24 (66.74-84.14)	0.1338
R3	0.845 (0.759-0.909)	0.0311*	100 (90.98-100)	0.0313*	27.42 (16.85-40.23)	<0.0001*	46.43 (42.65-50.25)	0.0012*	100 /	0.1696	55.45 (45.22-65.34)	<0.0001*
R4	0.825 (0.737-0.894)	0.0141*	97.44 (86.52-99.94)	0.0625	48.39 (35.50-61.44)	<0.0001*	54.29 (48.14-60.31)	0.0169*	96.77 (80.99-99.53)	0.2372	67.33 (57.28-76.33)	0.0015*
R5	0.801 (0.710-0.874)	0.0035*	100 (90.98-100)	0.0313*	12.90 (5.74-23.85)	<0.0001*	41.94 (39.62-44.29)	0.0002*	100 /	0.3437	46.53 (36.55-56.73)	<0.0001*

DL, deep learning; BI-RADS, Breast Imaging Reporting and Data System; AUC, area under the receiver operating characteristic curve; PPV, positive predictive value; NPV, negative predictive value; ACC, accuracy; CI, confidence interval; SPro, prospective BI-RADS assessment; R, radiologist.

P value, comparison diagnostic performance with DL model.

**P* values show statistical difference.

Supplementary Table 4

Performance metrics for the DL model versus the prospective BI-RADS assessment and the five radiologists in the comparison set from the external test sets

	AUC (95%CI)	*P* value	Sensitivity (95%CI)	*P* value	Specificity (95%CI)	*P* value	PPV (95%CI)	*P* value	NPV (95%CI)	*P* value	ACC	*P* value
DL	0.930 (0.861-0.971)		93.89 (83.13-98.72)		80.39 (66.88-90.18)		82.14 (72.43-88.96)		93.18 (81.91-97.63)		87.00 (78.80-92.89)	
Pro	0.979 0.929 to 0.997	0.0284*	100 (92.75-100)	0.2500	49.02 (34.75-63.40)	0.0009*	65.33 (59.02-71.15)	0.0337*	100 /	0.1851	74.00 (64.27-82.26)	0.0146*
R1	0.941 (0.875-0.978)	0.6575	95.92 (86.02-99.50)	1.0000	70.59 (56.17-82.51)	0.1797	75.81 (67.11-82.79)	0.4024	94.74 (82.08-98.61)	0.7706	83.00 (74.18-89.77)	0.4240
R2	0.894 (0.817-0.947)	0.2130	97.96 (89.15-99.95)	0.6250	39.22 (25.84-53.89)	<0.0001*	60.76 (55.31-65.96)	0.0080*	95.24 (73.61-99.31)	0.7489	68.00 (57.92-76.98)	0.0005*
R3	0.863 (0.780-0.924)	0.0522	100 (92.75-100)	0.2500	13.73 (5.70-26.26)	<0.0001*	52.69 (49.95-55.41)	0.0003*	100 /	0.4808	56.00 (45.72-65.92)	<0.0001*
R4	0.759 (0.663-0.839)	0.0002*	89.80 (77.77-96.60)	0.6875	45.10 (31.13-59.66)	0.0001*	61.11 (54.63-67.22)	0.0101*	82.14 (65.52-91.76)	0.1491	67.00 (56.88-76.08)	0.0002*
R5	0.768 (0.673-0.847)	0.0004*	95.92 (86.02-99.50)	1.0000	23.53 (12.79-37.49)	<0.0001*	54.65 (50.59-58.65)	0.0008*	85.71 (58.59-96.22)	0.3900	59.00 (48.71-68.74)	<0.0001*

DL, deep learning; BI-RADS, Breast Imaging Reporting and Data System; AUC, area under the receiver operating characteristic curve; PPV, positive predictive value; NPV, negative predictive value; ACC, accuracy; CI, confidence interval; Pro, prospective BI-RADS assessment; R, radiologist.

P value, comparison diagnostic performance with DL model.

[*]*P* value s show statistical difference.

Supplementary Table 5 Performance metrics for the five radiologists with and without model assistance in the comparison set

	AUC (95%CI)	*P* value	Sensitivity (95%CI)	*P* value	Specificity (95%CI)	*P* value	PPV (95%CI)	*P* value	NPV (95%CI)	*P* value	ACC	*P* value
Radiologists with DL assistance method one												
R1	0.955 (0.916-0.979)	0.0444[#]	98.86 (93.83-99.97)	0.2500	80.53 (72.02-87.39)	0.1671	79.82 (73.09-85.20)	0.3329	98.91 (92.82-99.84)	0.1593	88.56 (83.33-92.61)	0.0525
R2	0.909 (0.861-0.945)	0.4608	94.32 (87.24-98.13)	0.3750	79.65 (71.04-86.64)	<0.0001[#]	78.30 (71.40-83.91)	0.0048[#]	94.74 (88.43-97.70)	0.5603	86.07 (80.50-90.54)	<0.0001[#]
R3	0.853 (0.797-0.899)	0.5581	98.86 (93.83-99.97)	1.0000	47.79 (38.30-57.39)	<0.0001[#]	59.59 (55.25-63.79)	0.0768	98.18 (88.40-99.74)	0.5089	70.15 (63.31-76.38)	<0.0001[#]
R4	0.837 (0.778-0.885)	0.0005[#]	97.73 (92.03-99.72)	0.1250	65.49 (55.96-74.18)	<0.0001[#]	68.80 (63.06-74.02)	0.0625	97.37 (90.33-99.32)	0.0668	79.60 (73.36-84.95)	<0.0001[#]
R5	0.796 (0.733-0.849)	0.0160[#]	96.59 (90.36-99.29)	1.0000	47.79 (38.30-57.39)	<0.0001[#]	59.03 (54.60-63.32)	0.0497[#]	94.74 (85.34-98.24)	0.5337	69.15 (62.27-75.46)	<0.0001[#]
Radiologists with DL assistance method two												
R1	0.930 (0.886-0.961)	0.7264	95.46 (88.77-98.75)	1.0000	81.42 (73.01-88.11)	0.0574	80.00 (73.06-85.51)	0.3214	95.83 (89.79-98.37)	0.9001	87.56 (82.19-91.79)	0.0963
R2	0.937 (0.894-0.967)	0.0007[#]	98.86 (93.83-99.97)	1.0000	69.91 (60.57-78.18)	0.0005[#]	71.90 (65.87-77.23)	0.0749	98.75 (91.81-99.82)	0.4099	82.59 (76.62-87.56)	0.0003[#]
R3	0.936 (0.893-0.966)	0.0002[#]	97.73 (92.03-99.72)	0.5000	69.91 (60.57-78.18)	<0.0001[#]	71.67 (65.59-77.05)	0.0002[#]	97.53 (90.89-99.36)	0.4392	82.09 (76.08-87.13)	<0.0001[#]
R4	0.880 (0.827-0.921)	0.0040[#]	98.86 (93.83-99.97)	0.0625	54.87 (45.23-64.25)	0.1078	63.04 (58.17-67.67)	0.3658	98.41 (89.76-99.77)	0.0425[#]	74.13 (67.50-80.04)	0.0161[#]
R5	0.899 (0.849-0.937)	0.0001[#]	97.73 (92.03-99.72)	1.0000	58.41 (48.76-67.61)	<0.0001[#]	64.66 (59.47-69.53)	0.0036[#]	97.06 (89.26-99.24)	0.2263	75.62 (69.08-81.39)	<0.0001[#]

AUC, area under the receiver operating characteristic curve; PPV, positive predictive value; NPV, negative predictive value; ACC, accuracy; CI, confidence interval; DL, deep learning; R, radiologist.

The *P* values are that of radiologists with DL assistance vs. radiologists without DL assistance.

[#]*P* values show statistical difference.

Supplementary Table 6

Comparison of the diagnostic performance between radiologists with the assistance of method one and method two in comparison set

AUC, area under the receiver operating characteristic curve; PPV, positive predictive value; NPV, negative predictive value; ACC, accuracy; R, radiologist; All, all the five radiologists; Ex, experienced radiologists, Inex, inexperienced radiologists.

P values are that of radiologists with model assistance-method one vs. radiologists with model assistance-method two and * *P* values show statistical difference.

	AUC, *P* value	Sensitivity, *P* value	Specificity, *P* value	PPV, *P* value	NPV, *P* value	ACC, *P* value
R1	0.0470*	0.2500	1.0000	0.9733	0.1907	0.7744
R2	0.0154*	0.1250	0.0010*	0.2682	0.1473	0.1185
R3	0.0002*	1.0000	<0.0001*	0.1936	0.8005	0.0002*
R4	0.0890	1.0000	0.0227*	0.3267	0.6743	0.0433*
R5	0.0003*	1.0000	0.0576	0.3360	0.5111	0.0470*
All	<0.0001*	0.7905	0.1915	0.5037	0.6076	0.1587
Ex	0.9290	1.0000	0.0414*	0.3937	0.8384	0.1221
Inex	<0.0001*	1.0000	0.0134*	0.2286	0.6116	0.0129*

Supplementary Table 7

The results of the model tested on the internal and external test sets for 10 times

ITC, internal test cohort; ETC, external test cohort; AUC, area under the receiver operating characteristic curve; SENS, sensitivity; SPEC, specificity; PPV, positive predictive value; NPV, negative predictive value; ACC, accuracy; MCC, Matthews correlation coefficient; SD, standard deviation.

ITC	AUC	SENS	SPEC	PPV	NPV	ACC	F1	MCC
1	0.907	81.99	83.94	72.93	89.83	83.26	0.772	0.643
2	0.909	80.75	84.59	73.45	89.27	83.26	0.769	0.640
3	0.906	81.99	84.26	73.33	89.86	83.48	0.774	0.647
4	0.909	82.61	84.26	73.48	90.18	83.69	0.778	0.652
5	0.907	81.34	83.28	71.98	89.44	82.62	0.764	0.630
6	0.913	82.61	85.25	74.72	90.28	84.34	0.785	0.664
7	0.902	79.50	83.93	72.32	88.58	82.40	0.757	0.622
8	0.913	80.75	84.26	73.03	89.24	83.05	0.767	0.636
9	0.905	81.37	82.95	71.58	89.40	82.40	0.762	0.626
10	0.906	80.12	83.28	71.67	88.81	82.19	0.757	0.619
Average	0.908	81.48	83.97	72.85	89.57	83.11	0.769	0.639
SD	0.003	1.033	0.688	0.976	0.553	0.675	0.009	0.014
ETC	AUC	SENS	SPEC	PPV	NPV	ACC	F1	MCC
1	0.921	88.84	84.29	85.92	87.50	86.65	0.874	0.733
2	0.918	88.35	82.20	84.26	86.74	85.39	0.863	0.708
3	0.912	89.32	83.25	85.19	87.85	86.40	0.872	0.728
4	0.912	87.86	82.20	84.19	86.26	85.14	0.860	0.703
5	0.915	88.35	82.72	84.65	86.81	85.64	0.865	0.713
6	0.919	88.84	84.29	85.92	85.19	86.65	0.874	0.733
7	0.916	86.41	84.29	85.58	87.50	85.40	0.860	0.707

8	0.915	89.32	83.25	85.19	87.85	86.40	0.872	0.728
9	0.911	88.84	83.25	85.12	87.36	86.15	0.869	0.723
10	0.919	88.35	83.25	85.05	86.89	85.89	0.867	0.718
Average	0.916	88.48	83.34	85.14	87.03	86.01	0.868	0.720
SD	0.003	0.850	0.798	0.607	0.817	0.557	0.005	0.011

Supplementary Table 8

The performance comparison between VGG-19 and other models

AUC, area under the receiver operating characteristic curve; PPV, positive predictive value; NPV, negative predictive value.

	AUC	Sensitivity	Specificity	PPV	NPV	Accuracy
ResNet-18	0.874	81.99%	79.02%	67.35%	89.26%	80.04%
ResNet-50	0.916	81.99%	82.95%	71.74%	89.72%	82.62%
EfficientNetV2-S	0.910	81.99%	**84.82%**	74.16%	89.93%	83.91%
VGG-16	0.931	**88.82%**	80.66%	70.79%	93.18%	83.48%
VGG-19	**0.933**	**88.82%**	83.93%	**74.48%**	**93.43%**	**85.62%**

Supplementary Table 9

Intra-observer variability in the BI-RADS assessment

BI-RADS, Breast Imaging Reporting and Data System; R, radiologist; CI, confidence interval.

Radiologist	Kappa value (95%CI)
R1	0.81(0.76-0.85)
R2	0.77(0.72-0.82)
R3	0.49(0.42-0.56)
R4	0.46(0.38-0.54)
R5	0.49(0.41-0.57)
